# Exploring the Association Between Self-Reported Asthma Impact and Fitbit-Derived Sleep Quality and Physical Activity Measures in Adolescents

**DOI:** 10.2196/mhealth.7346

**Published:** 2017-07-25

**Authors:** Jiang Bian, Yi Guo, Mengjun Xie, Alice E Parish, Isaac Wardlaw, Rita Brown, François Modave, Dong Zheng, Tamara T Perry

**Affiliations:** ^1^ Department of Health Outcomes and Policy University of Florida Gainesville, FL United States; ^2^ Department of Computer Science University of Arkansas at Little Rock Little Rock, AR United States; ^3^ Arkansas Children’s Research Institute Department of Pediatrics University of Arkansas for Medical Sciences Little Rock, AR United States; ^4^ Department of Electrical and Computer Engineering University of Florida Gainesville, FL United States; ^5^ Department of Pediatrics University of Arkansas for Medical Sciences Little Rock, AR United States; ^6^ Arkansas Children's Hospital Arkansas Children's Research Institute Little Rock, AR United States

**Keywords:** mobile health, mHealth, asthma, Fitbit, physical activity, sleep, sleep quality

## Abstract

**Background:**

Smart wearables such as the Fitbit wristband provide the opportunity to monitor patients more comprehensively, to track patients in a fashion that more closely follows the contours of their lives, and to derive a more complete dataset that enables precision medicine. However, the utility and efficacy of using wearable devices to monitor adolescent patients’ asthma outcomes have not been established.

**Objective:**

The objective of this study was to explore the association between self‑reported sleep data, Fitbit sleep and physical activity data, and pediatric asthma impact (PAI).

**Methods:**

We conducted an 8‑week pilot study with 22 adolescent asthma patients to collect: (1) weekly or biweekly patient‑reported data using the Patient-Reported Outcomes Measurement Information System (PROMIS) measures of PAI, sleep disturbance (SD), and sleep‑related impairment (SRI) and (2) real-time Fitbit (ie, Fitbit Charge HR) data on physical activity (F-AM) and sleep quality (F‑SQ). To explore the relationship among the self-reported and Fitbit measures, we computed weekly Pearson correlations among these variables of interest.

**Results:**

We have shown that the Fitbit-derived sleep quality F-SQ measure has a moderate correlation with the PROMIS SD score (average *r*=−.31, *P*=.01) and a weak but significant correlation with the PROMIS PAI score (average *r*=−.18, *P*=.02). The Fitbit physical activity measure has a negligible correlation with PAI (average *r*=.04, *P*=.62).

**Conclusions:**

Our findings support the potential of using wrist-worn devices to continuously monitor two important factors—physical activity and sleep—associated with patients’ asthma outcomes and to develop a personalized asthma management platform.

## Introduction

Childhood morbidity due to asthma is particularly high among adolescent patients [1]. Adolescents have a higher prevalence of asthma compared with younger children [2]. They are at a higher risk for adverse health outcomes and are more likely to experience an exacerbation requiring hospitalization, intubation, or cardiopulmonary resuscitation [3]. The adolescent years are critically important for a person’s physical and psychological development, which in turn requires sufficient sleep [4] and physical activity; however, both sleep and physical activity can be significantly constrained by asthma [5,6]. Conversely, asthma symptoms can worsen because of sleep problems [7] and strenuous exercise [8]. Nevertheless, regular physical activity is an important component of pediatric asthma management [9]. Physical activity has also been associated with a decrease in the severity of the symptoms of asthma and an improvement in the quality of life among children with asthma [10-12]. Therefore, unveiling the relationship between asthma, sleep patterns [13], and physical activity can potentially aid health care providers when counseling adolescents on asthma self-management, improving quality of life, and facilitating the discovery of more effective interventions for asthma.

Prior studies have reported a relationship between asthma and sleep among adolescents [14,15]. Uncontrolled asthma is associated with impaired nighttime sleep, daytime sleepiness, and health-related quality of life. Compared with healthy children, children with asthma are more likely to report sleep problems due to greater increase in airway resistance during nighttime sleep, which in turn leads to exacerbated asthma symptoms such as coughing and wheezing [16]. However, there are substantial barriers to examining the relationship between asthma and sleep patterns. Such studies usually need to be carried out in a special environment (for capturing sleep patterns [17-20]) and over a relatively long time (to accumulate enough data), which incurs high costs. Specialized and expensive equipment is typically needed to collect sleep pattern data, and participants usually have to stay in an unfamiliar sleep study facility [19,20].

However, the advent of smart wearables (eg, wrist-worn mobile devices such as the Fitbit wristband) opens a new horizon for continuous, unobtrusive, and cost-effective data collection [21-24]. The concept is compelling: smart wearables provide the opportunity to monitor patients more comprehensively, to track patients in a fashion that more closely follows the contours of their lives, and to derive a more complete dataset that enables precision medicine [25]. Although multipurpose wrist‑worn devices present some limits with respect to accuracy, they are reasonable options for near-continuous data monitoring [26]. It has been reported that these devices have acceptable reliability and validity when used to measure physical activity and sleep in adults [27]. Specifically, for physical activity tracking, it has been reported [28-30] that these devices have acceptable accuracy at various speeds, attachment sites, and in both lab and real-world settings, particularly for step counting. More recently, it was shown that age and pathological gait changes might affect the accuracy of step counting devices. However, this does not apply to our specific study [31]. By wearing wrist-worn devices, long-term motion and biological data that patients generate in their living environment can be collected through device built-in sensors (eg, accelerometer, gyroscope, and heart rate sensors) in a user-transparent manner. Furthermore, there is a growing interest in using sensor-based observations through smartphones and wearables as a novel way to collect health information, accelerated as a part of the precision medicine initiative [25]. Technologies such as wearables will provide new opportunities for patient engagement and care delivery that will generate precision interventions, which will ultimately enhance clinical outcomes and reduce health care costs [32].

Although asthma is a serious health problem among adolescents, the utility and efficacy of using wearable devices to monitor adolescent patients’ asthma outcomes have not been established. In this pilot study, we aimed to explore the association among patient‑reported sleep data, Fitbit sleep and physical activity data, and pediatric asthma outcomes. We collected patient‑reported outcomes (PROs) on pediatric asthma impact (PAI), sleep disturbance (SD), and sleep‑related impairment (SRI) [33,34], as well as Fitbit-derived sleep and physical activity measures from 22 adolescents with asthma. The goals of the study were to explore the association between (1) asthma outcomes and Fitbit-derived sleep and physical activity measures and (2) patient‑reported sleep measures and Fitbit-derived sleep measures.

## Methods

### Study Design

**Figure 1:**
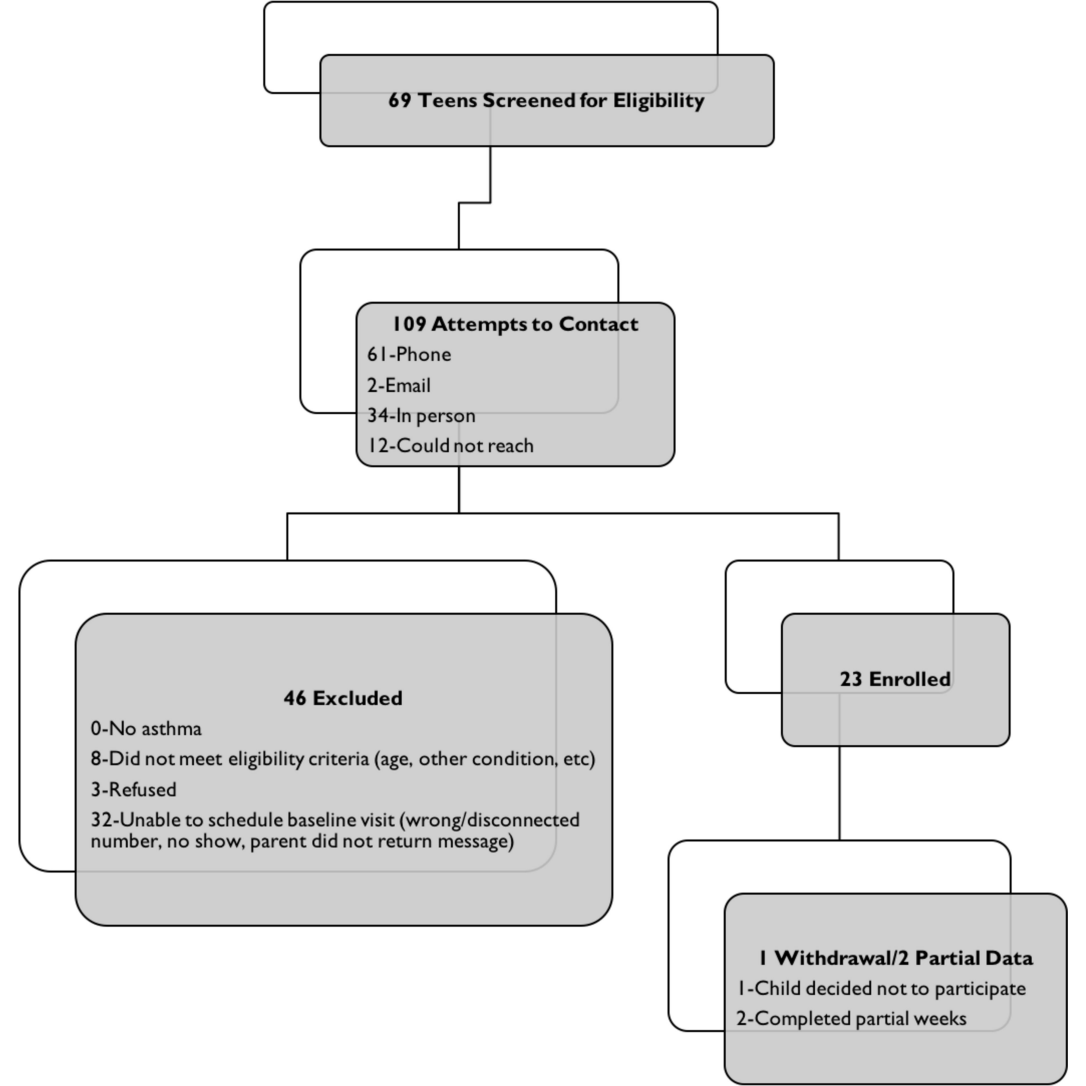
Cumulative screening and enrollment summary.

We conducted an 8-week pilot study (trial registration number NCT02556567) and recruited a convenience sample of adolescent asthma patients according to their medical chart from the Arkansas Children’s Hospital subspecialty asthma clinics. Patients were screened for eligibility after their outpatient visits, where the physician discussed the study briefly with the patient and the caregiver, and a research nurse carried out the rest of the recruitment activities. Inclusion criteria required the participants to: (1) be in the age group of 14 to 17 years with physician-diagnosed persistent asthma and (2) to have access to a smartphone or a computer that was compatible with the Fitbit app. Informed consent and assent were obtained from all participants and their caregivers, respectively, during the baseline visit. Figure 1 shows the cumulative screening and enrollment summary for the study. Of the 23 enrolled participants, 1 participant withdrew from the study at the beginning of the study, and 2 did not complete all 8 weeks of the study procedures. Our data analysis excluded the participant who withdrew, resulting in 22 adolescents in our data analysis. There were 17 participants with complete data (8 weeks). We also included participants with partial data where 2 participants had 7 weeks complete, one with 6 weeks, one with 4 weeks, and one with 3 weeks.

**Figure 2:**
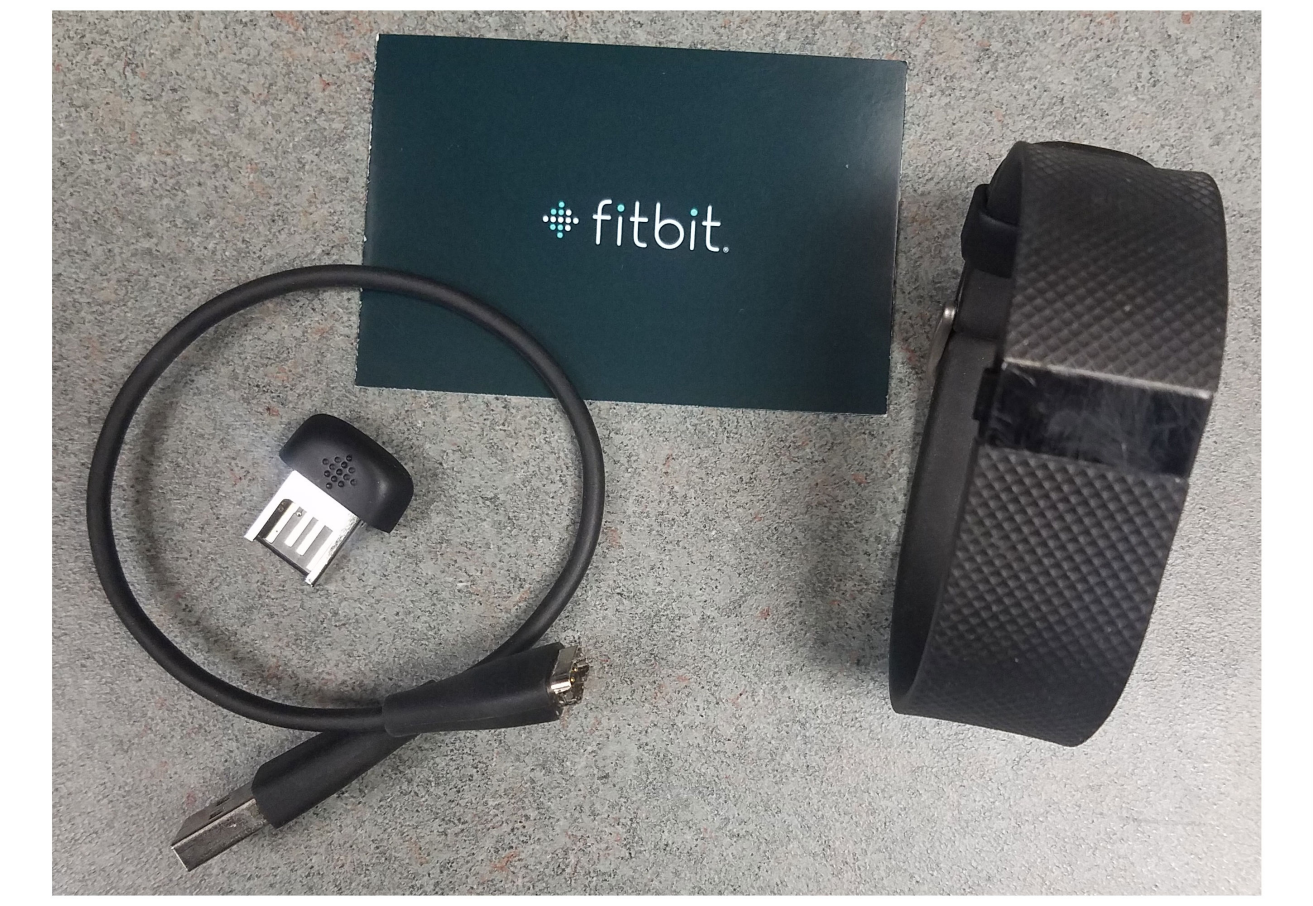
Fitbit Charge HR wristband.

During the baseline visit, all participants had their height and weight measured and completed a baseline survey to collect demographic data (eg, gender, race, and ethnicity), socioeconomic status (eg, household income), insurance information, baseline level of asthma control according to the national guidelines, medication history, and baseline PROs (ie, PAI, SD, and SRI [33]). The caregivers of the participants were also present during the baseline visit and helped answer some of the questions (eg, income). The research nurse also showed the participants how to navigate through the Fitbit user interface during the baseline visit. During the study, all participants were given a Fitbit Charge HR wristband (Figure 2) and instructed to wear the wristband at all times (24 hours every day) during the 8-week study period, except for when the device was being charged. Participants’ biological data were collected in real time and passively without user interactions with the Fitbit devices and then uploaded to the Fitbit/Fitabase servers [35]. In addition, participants were asked to complete Web-based surveys to collect the PROs. The asthma impact survey was administered weekly and the two sleep‑related surveys were administered biweekly to avoid survey fatigue. As an incentive for participation, participants received US $10 for completion of weekly surveys and synchronization of Fitbit data. Study procedures were approved by the institutional review board of the University of Arkansas for Medical Sciences.

### Measures

### Fitbit Measures

The Fitbit Charge HR wristband can collect a wide range of physical and biological data, including physical activity (eg, calories burned, steps, distance, and heart rate) and sleep data (eg, time in bed and awakenings count). We used two summary measures derived from Fitbit data, Active Minutes (F-AM) and Sleep Quality (F-SQ) score. F-AM was defined as daily minutes of moderate and vigorous activity, and F-SQ score was defined as the ratio of minutes asleep to minutes in bed.

### Patient-Reported Outcomes

PROs were collected using the Patient-Reported Outcomes Measurement Information System (PROMIS) instruments [33,36], which included pediatric asthma impact (PAI; short form 8a), sleep disturbance (SD; short form 6a), and sleep-related impairment (SRI; short form 8a). These instruments were previously validated among adolescents [37,38].

The PAI short form 8a contains 8 questions asking whether the patients had asthma-specific symptoms such as cough, wheeze, shortness of breath, and avoidance of triggers in the past 7 days. The responses are on an ordinal scale going from Never (0 point) to Almost Always (4 points). A higher PAI score means worse asthma outcomes. Prior studies have reported good Cronbach alpha (>.8) for the PAI short form 8a [39].

The SD short form 6a contains 6 questions that evaluate self‑reported perceptions of sleep quality, sleep depth, and restoration associated with sleep in the past 7 days. For example, one question asks “I had difficulty falling asleep...,” and the responses include the following: Not at all (5 points), A little bit, Somewhat, Quite a bit, and Very much (1 point). A higher SD score means less sleep disturbance. The Cronbach alpha for the SD short form 6a has been reported to be good (>.8; [40-43]).

The SRI short form 8a contains 8 questions that evaluate self‑reported perceptions of alertness, sleepiness, and tiredness during usual waking hours and the perceived functional impairments during wakefulness associated with sleep problems or impaired alertness in the past 7 days. For example, one question asks “I had a hard time getting things done because I was sleepy…,” and the responses include the following: Not at all (5 points), A little bit, Somewhat, Quite a bit, and Very much (1 point). A higher SRI score means less sleep-related impairment. Prior studies have also reported good Cronbach alpha (>.8) for the SRI short form 8a [41-43].

All PROMIS measures are reported on a T-score scale (mean= 50, SD=10). This allows us to easily compare various PROMIS scores across scales, even if the original scores do not have identical variances or are measured on different scales [33]. For each instrument, we computed the raw scores by adding up scores from each question and converted the raw scores to T-scores for data analysis. All instruments are presented in Multimedia Appendices 1-.

### Statistical Analysis

We first calculated descriptive statistics to summarize the participants’ characteristics. We then computed weekly Spearman rank-order correlations among the variables of interest, which included pediatric asthma outcome PAI, Fitbit-derived measures F‑SQ and F‑AM, and self-reported sleep measures SD and SRI. We used the following guidelines for interpreting correlation coefficients: <.3=poor, .3 to .7=moderate, and >.7=excellent. All analyses were conducted with Statistical Analysis Software (SAS) version 9.4 (SAS Institute Inc).

## Results

The participants’ characteristics are summarized in Table 1. The average age of our participants was 15.5 years (SD 1.1). There were 12 boys (55%) and 10 girls (45%). The race distributions of the participants were 12 whites (55%), 7 African Americans (32%), and 3 other races (13%). The majority (64%; 14 participants) of the participants were covered by state insurance, 7 (32%) were covered by private insurance, and 1 (4%) was uninsured. The annual household income was below $60,000 for most of the participants.

**Table 1:** Participants’ characteristics.

Characteristic		n (%) or mean (SD) (N=22)
**Age in years, mean (SD)**		15.5 (1.1)
**Sex, n (%)**			
	Female	10 (45)
	Male	12 (55)
**Race, n (%)**			
	White	12 (55)
	Black	7 (32)
	Other	3 (13)
**Insurance, n (%)**			
	Private	7 (32)
	State	14 (64)
	Uninsured	1 (4)
**Household income in US dollars, n (%)**			
	<15,000	2 (9)
	15,000-60,000	10 (45)
	>60,000	5 (23)
	Missing	5 (23)

**Figure 3:**
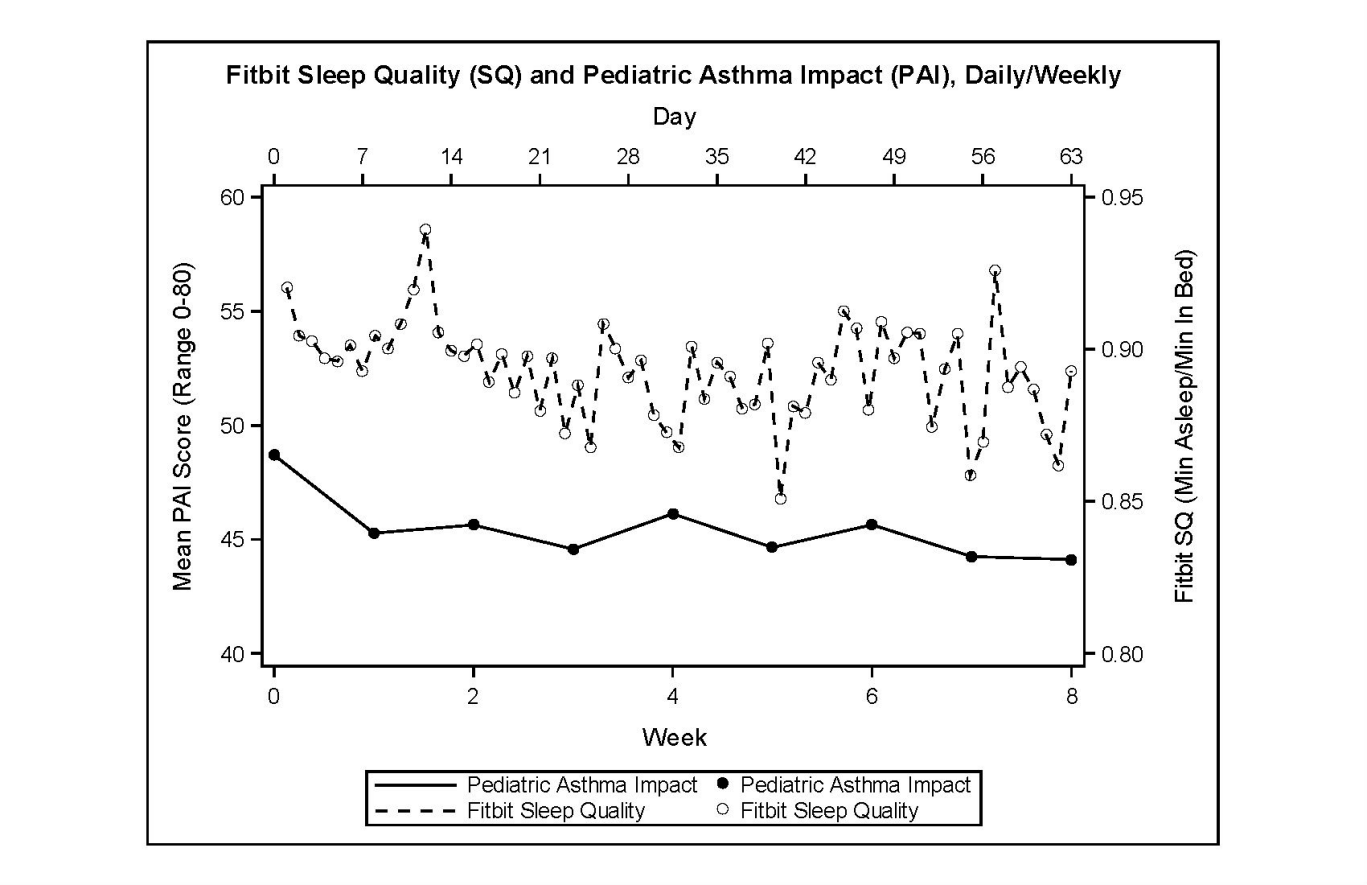
Fitbit sleep quality and pediatric asthma impact.

**Figure 4:**
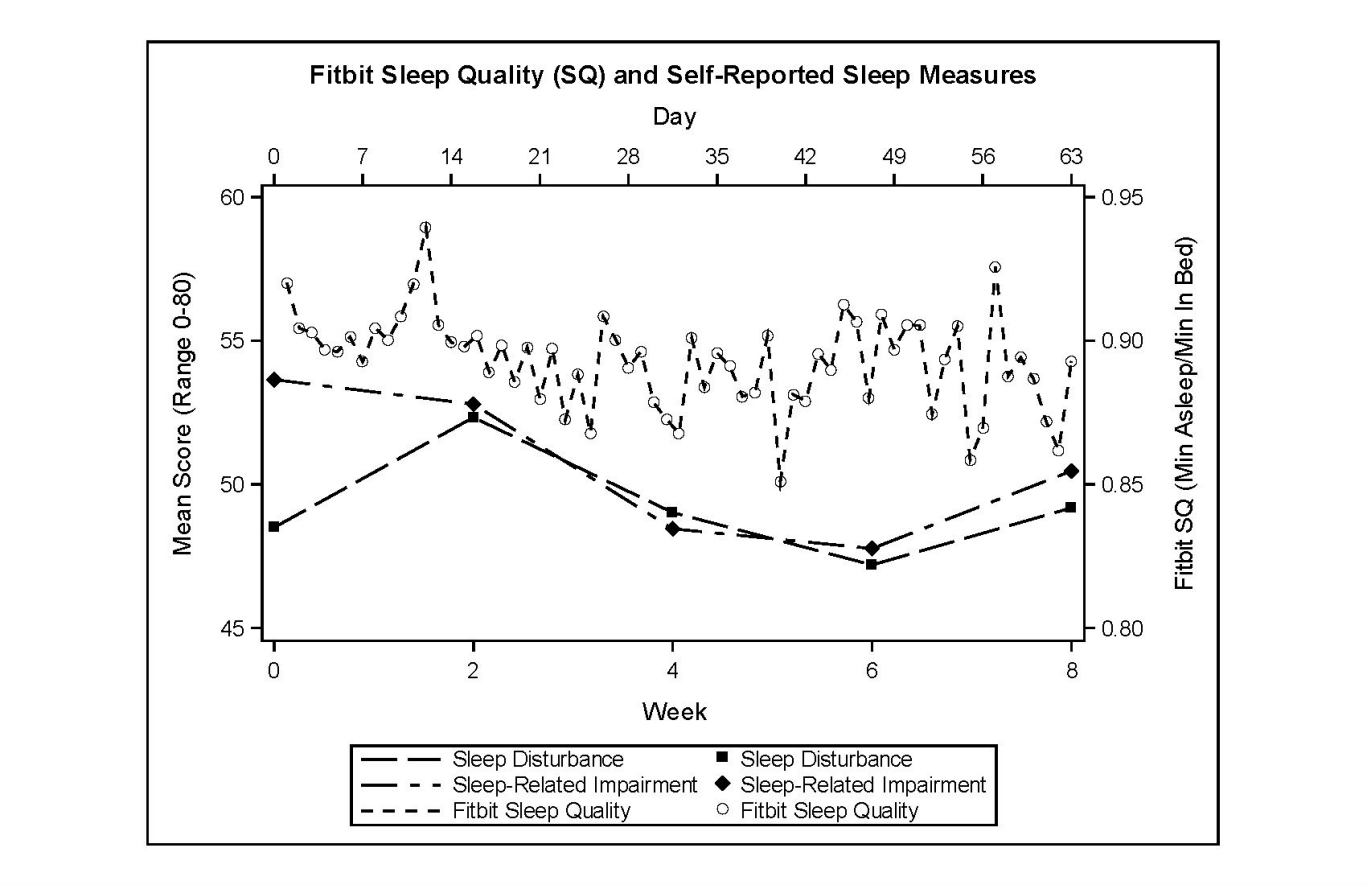
Fitbit sleep quality and self-reported sleep measures.

The variables of interest are summarized in Table 2 and to visually examine how the variables of interest changed across the study period, we plotted the Fitbit SQ score with the PAI score (Figure 3) and with the self‑reported SD and SRI scores (Figure 4) across the 8 weeks of our study. As seen in Figure 3, the average weekly PAI score varied between 44.12 and 48.70, with an average of 45.44. The average daily Fitbit SQ score varied between 0.85 and 0.94, with an average of 0.89. As seen in Figure 4, the biweekly self-reported SD score varied between 47.19 and 52.33, with an average of 49.24. The biweekly self-reported SRI score varied between 47.76 and 53.64, with an average of 50.62.

**Table 2:** Patient-reported outcomes and Fitbit measure statistics.

Measure	Median	Mean	Standard deviation	Skewness	Kurtosis
PAI^a^	46.50	44.99	10.45	0.15	−1.12
SD^b^	49.00	49.49	9.27	0.03	−0.36
SRI^c^	50.30	49.93	11.27	0.07	−0.97
F-AM^d^	0.00	10.59	21.59	4.08	24.21
F-SQ^e^	0.93	0.90	0.12	−2.69	6.37

^a^PAI: pediatric asthma impact.

^b^SD: sleep disturbance.

^c^SRI: sleep-related impairment.

^d^F-AM: Fitbit-Active Minutes.

^e^F-SQ: Fitbit-Sleep Quality.

We summarized the correlations among the variables of interest by week in Table 3. Our results showed that across the study period, higher PAI score was weakly associated with a lower SQ score. The weekly F‑SQ **–**PAI correlation ranged from −.48 to .19, with the average being −.18. On the other hand, the Fitbit physical activity measure (F‑AM) did not have a significant correlation with PAI, where the weekly F‑AM **–**PAI correlation ranged from a weak negative association to moderate positive association (−.26 to .39), suggesting a potentially complex relationship between these two variables. There was a moderate to strong positive association between PAI and the two sleep measures, SD and SRI, with the average weekly correlation being .71 and .64 for PAI **–**SD and PAI **–**SRI, respectively. Finally, we observed that as F-SQ increased, there was a moderate decrease in the PRO sleep disturbance (average weekly correlation=−.31).

**Table 3:** Spearman rank-order correlations among the variables of interests across the study period.

Variable Pairs	Week (*P* value)								
	1	2	3	4	5	6	7	8	Average
PAI^a^–F-SQ^b^	.04 (.88)	−.48 (.02)	−.57 (.01)	−.07 (.78)	−.07 (.77)	.19 (.41)	−.17 (.51)	−.21 (.44)	−.18 (.02)
PAI–F-AM^c^	−.09 (.70)	−.01 (.97)	.09 (.70)	.04 (.86)	−.26 (.28)	−.02 (.94)	.39 (.09)	.10 (.69)	.04 (.62)
PAI–SD^d^		.75 (<.01)		.62 (<.01)		.71 (<.01)		.75 (<.01)	.71 (<.01)
PAI–SRI^e^		.53 (.01)		.74 (<.01)		.67 (<.01)		.59 (<.01)	.64 (<.01)
F-SQ–SD		−.63 (<.01)		−.09 (.72)		−.16 (.49)		−.37 (.17)	−.31 (.01)
F-SQ–SRI		−.32 (.15)		−.09 (.70)		.08 (.73)		−.05 (.86)	−.08 (.50)

^a^PAI: pediatric asthma impact.

^b^F-SQ: Fitbit-Sleep Quality.

^c^F-AM: Fitbit-Active Minutes.

^d^SD: sleep disturbance.

^e^SRI: sleep-related impairment.

## Discussion

### Principal Findings

In this pilot study, we have shown that the Fitbit-derived sleep quality measure F‑SQ has a weak but significant inverse association with the PROMIS PAI score. In addition, F‑SQ has a moderate inverse correlation with the PROMIS SD measure. Overall, our study suggests that there is a potential association between Fitbit measures or other wrist-worn activity tracking devices and asthma outcomes. Further studies, with more power, would be able to determine whether Fitbit measures could predict the symptoms of pediatric asthma.

### Fitbit Sleep Quality and PROMIS Sleep Measures

The Fitbit-derived F‑SQ score has a moderate negative correlation with the SD measure but no correlation with the SRI measure. One potential reason is that the F‑SQ score is the ratio of minutes asleep over minutes spent in bed, which is an indicator of sleep quality. Therefore, it is conceptually more similar to the SD measure, which evaluates perceived difficulties with getting to sleep or staying asleep. On the other hand, the SRI measure is not a measure of sleep quality. SRI is concerned with functional impairments, such as alertness, sleepiness, and tiredness, associated with sleep problems during usual waking hours.

### Fitbit Physical Activity and Asthma Impact

The Fitbit physical activity measure was not associated with the asthma impact despite what the existing literature suggests (ie, physical activity is associated with asthma outcomes [10,12]). There are several potential reasons that we did not find such association, which include the following: (1) our pilot study was not sufficiently powered and (2) the relationship between physical activity and asthma is more complex. For example, to capture exercise-induced bronchoconstriction, also called exercise-induced asthma, asthmatic events will likely need to be captured in real time in addition to weekly patient-reported asthma impact. Furthermore, the effects of physical activity on patients’ general asthma outcomes are long term and may not be observed in an 8-week study. In short, more studies and data are needed to further explore the relationship between Fitbit data and patient‑reported measures.

The success of the pilot study demonstrates the utility of passively collected sensory data for monitoring factors related to asthma, and that ultimately, these data could be potentially incorporated into a disease management tool. In our previous study [44], we pilot-tested a smartphone-—based mobile asthma action plans (mAAP) app and showed its acceptance among adolescents. Incorporating models for detecting asthma triggers into the mAAP app will allow us to build a mobile health (mHealth) platform that can give us a more complete picture of patient’s disease states and then provide them with real-time personalized asthma management strategies. Nevertheless, to achieve this goal, we shall further explore sources that would have data on other asthma triggers beyond physical activity and sleep. For example, allergens and irritants in the air are common asthma triggers that can be measured with particle sensors.

### Limitations

Due to the pilot nature of this project, our study has distinct limitations. First, with only 22 participants, the study was not adequately powered for complex statistical analyses. Therefore, influences from covariates, such as gender and race, on the outcome-predictor relationships were not considered. Second, the study participants were recruited as a convenience sample rather than as a random sample. It is therefore unknown whether the results from this study generalize well to other populations. Third, the PAI survey has a question about trouble sleeping at night that could explain the correlations between the PAI measure and our sleep measures. Last, the accuracy of using wearable devices for long-term monitoring of physical activity and sleep is suboptimal, and device dependent [26]. Future studies are warranted to further explore the use of different wearable devices to replicate our findings using Fitbit.

### Conclusions

Wrist-worn activity tracking devices such as Fitbit are associated with pediatric asthma PROs, and with further research, have the potential to closely monitor patients’ biological data and to help manage chronic diseases where sleep quality and physical activity are factors. Our long-term goal is to develop an mHealth platform with a wrist-worn device that can collect sensory data related to asthma triggers and create models that predict patients’ asthma outcomes with these factors, thereby providing personalized asthma management strategies to patients in real time.

## Appendix

Multimedia Appendix 1 Pediatric Asthma Impact short form 8a.

Multimedia Appendix 2 Sleep Disturbance short form 6a.

Multimedia Appendix 3 Sleep Related Impairment short form 8a.

## Abbreviations

F-AM: Fitbit-Active Minutes
F-SQ: Fitbit-Sleep Quality
mAAP: mobile asthma action plans
mHealth: mobile health
PAI: pediatric asthma impact
PRO: patient-reported outcome
PROMIS: Patient-Reported Outcomes Measurement Information System
SAS: Statistical Analysis Software
SD: sleep disturbance
SRI: sleep-related impairment
