# Supplementary figures and images for: Exploring the Association Between Self-Reported Asthma Impact and Fitbit-Derived Sleep Quality and Physical Activity Measures in Adolescents
Source: JMIR Mhealth Uhealth. 2017 Jul 25;5(7):e105. doi: 10.2196/mhealth.7346 (PMC5548986; doi:10.2196/mhealth.7346)

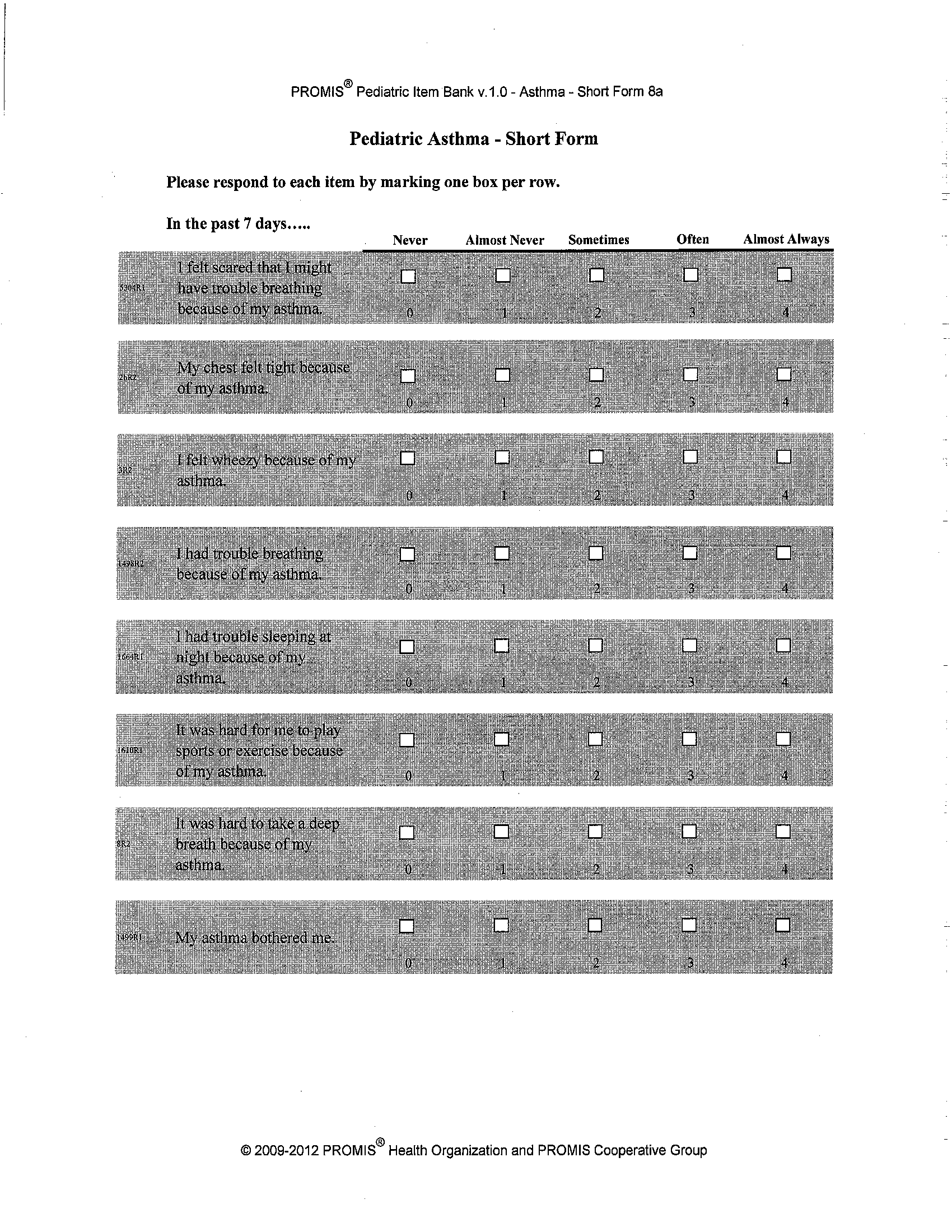

Supplement: Multimedia Appendix 1 [file mhealth_v5i7e105_app1.png]

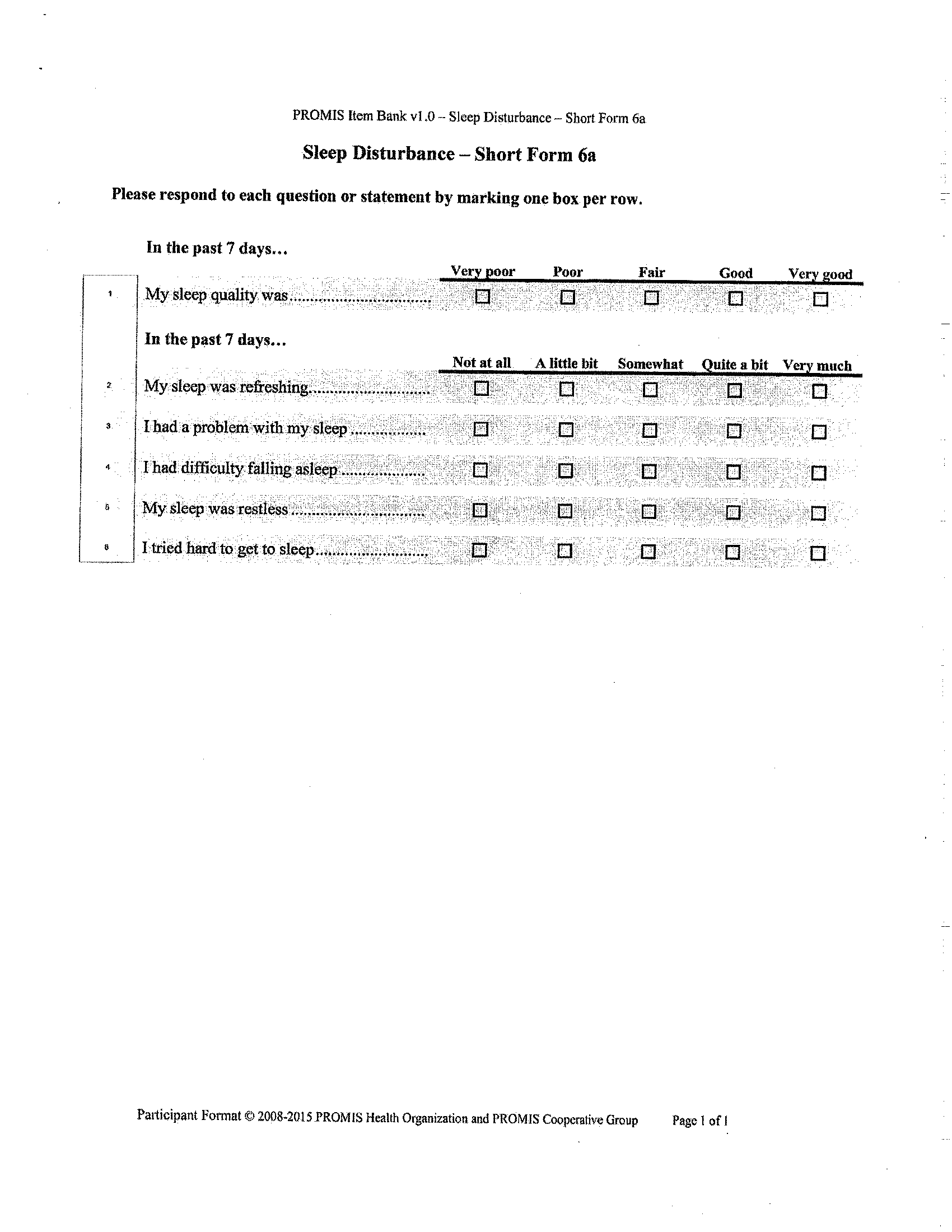

Supplement: Multimedia Appendix 2 [file mhealth_v5i7e105_app2.png]

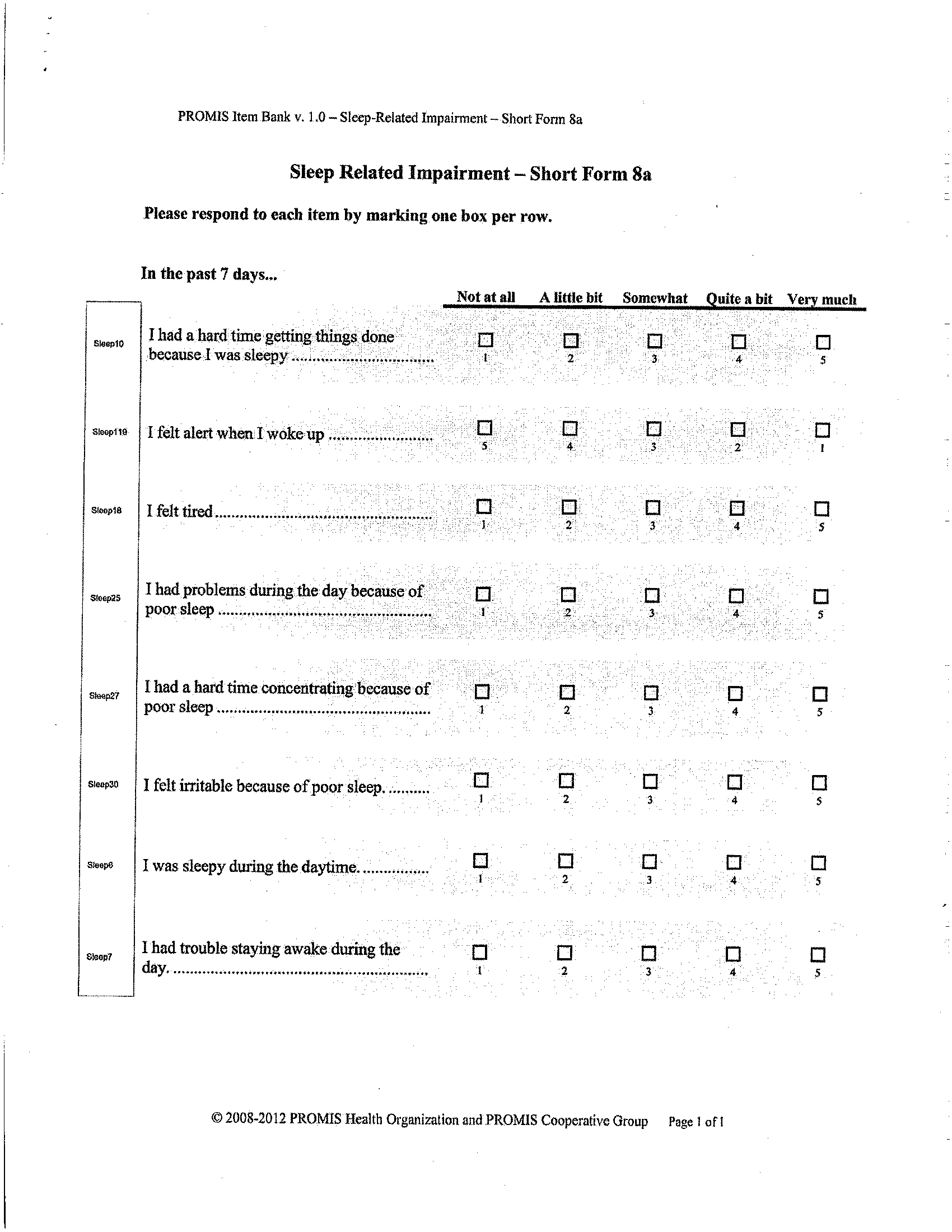

Supplement: Multimedia Appendix 3 [file mhealth_v5i7e105_app3.png]
